# Pharmacological inhibition of mTORC1 activity protects against inflammation-induced apoptosis of nucleus pulposus cells

**DOI:** 10.1590/1414-431X202010185

**Published:** 2021-03-15

**Authors:** Rigao Chen, Fei Yang, Yong Wang, Xinling Wang, Xiaohong Fan

**Affiliations:** 1 Department of Orthopedics, Hospital of Chengdu University of Traditional Chinese Medicine, Chengdu, China; 2 School of Clinical Medicine, Chengdu University of Traditional Chinese Medicine, Chengdu, China; 3 School of Basic Medical Sciences, Chengdu University of Traditional Chinese Medicine, Chengdu, China

**Keywords:** Intervertebral disc degeneration, Nucleus pulposus cells, mTORC1, Lactic acid, Apoptosis

## Abstract

Lumbar disc herniation is a common disease characterized by the degeneration of intervertebral discs (IVDs), accompanied by imbalance of metabolic and inflammatory homeostasis. Current studies establish that IVD degeneration is induced by increased apoptosis of nucleus pulposus (NP) cells. However, the underlying mechanisms of NP cell survival/apoptosis are not well elucidated. Here, we reveal a novel mechanism by which mTORC1 signaling controls NP cell survival through regulating metabolic homeostasis. We demonstrated that hyperactivated mTORC1 activity induced by inflammatory cytokines engenders the apoptosis of NP cells, whereas pharmacological inhibition of mTORC1 activity promotes NP cell survival. Using an integrative approach spanning metabolomics and biochemical approaches, we showed that mTORC1 activation enhanced glucose metabolism and lactic acid production, and therefore caused NP cell apoptosis. Our study identified mTORC1 in NP cells as a novel target for IVD degeneration, and provided potential strategies for clinical intervention of lumbar disc herniation.

## Introduction

Lumbar disc herniation (LDH) is a common disease characterized by the degeneration of intervertebral discs (IVDs), accompanied by imbalance of metabolic and inflammatory homeostasis (1,2). LDH progression often engenders lumbar radiculopathy due to the contact of extruded disc material or pressure on the thecal sac or lumbar nerve roots (2,3). The incidence of LDH within certain populations has been estimated to be more than 50% of asymptomatic adults (2,4). The underlying mechanism of LDH is not well elucidated. Non-operative and operative strategies are widely used for LDH interventions, including physical therapy, alternative medicine options, and surgical intervention (4,5). However, non-operative treatment is accompanied by LDH relapse, whereas operative surgery usually results in the impairment of lumbar intervertebral discs (5). Therefore, it is of immediate importance to investigate the pathological mechanisms of LDH to design novel therapeutic targets and strategies.

Degeneration of IVD occurs with dysregulated metabolism due to inflammation (6). Studies report that IVD degeneration coincides with decreased matrix production (7,8), increased degradative enzymes (9,10), pro-inflammatory cytokine expression (11,12), and ectopic cell proliferation and death (13,14). For example, leptin stimulates the proliferation of disc cells to form cell clusters and fibro-cartilaginous tissue, and thus contributes to IVD degeneration progression (15). On the other hand, dysregulation of intracellular pathways are also implicated in IVD degeneration by regulating cell apoptosis. Phosphatidylinositol 3-kinase (PI3K)/AKT pathway is reported to protect against IVD degeneration by suppressing the apoptosis of nucleus pulposus cells (NP cells) (14,16). Moreover, mTORC1 signaling that is downstream of PI3K/AKT also participates in NP cells apoptosis in IVD degeneration (17). Thus, there is an intimate link between mTORC1 signaling and IVD degeneration.

The mechanistic target of rapamycin (mTOR) is serine/threonine kinase, which includes mTORC1 and mTORC2 complex. mTORC1 is a master regulator of global cellular metabolism and thus controls cell growth, differentiation, apoptosis, and autophagy (18–21). Earlier studies suggest that selective interference of mTORC1 activity seems to protect against apoptosis of human disc cells, through regulating cell senescence and extracellular matrix (17). However, the mechanistic interplay between mTORC1 signaling and NP cell survival in IVD degeneration is not yet elucidated. In this study, we reveal a novel mechanism by which mTORC1 signaling controls NP cell survival. Hyper-activated mTORC1 activity induced by inflammatory cytokines increased apoptosis in NP cells, and blocking of mTORC1 activity promoted NP cell survival. Using an integrative approach spanning metabolomics and functional approaches, we demonstrated that mTORC1 signaling enhanced glucose metabolism by lactic acid production, and therefore caused NP cell apoptosis. Our study identified mTORC1 as a novel target for IVD degeneration, and provided potential strategies for LDH intervention.

## Material and Methods

### Cell culture

Human nucleus pulposus cells were cultured with 10% FBS (PAN, Germany) in basic DMEM medium (Gibco, USA), incubated under 5% CO_2_ at 37°C. Interleukin (IL)-1β (10 ng/mL, Sigma, USA), Torin1 (100 nM, Tocris, England), and lactate (100 mM, Machlin, England) were applied to NP cells for 24 h.

### Cell viability and apoptosis assay

Cell viability was detected by Cell Counting kit-8 (Dojindo China, China) assay. Briefly, NP cells were cultured in a density of 1,000 cells/well counting by the hemocytometer in 96-well plates. CCK-8 diluted at 1:10 with FBS-free DMEM medium was added to each well followed by 3 h under a temperature incubator at 37°C. Relative absorbance was measured on a microplate reader at 450 nm wavelength (Bio-Rad Laboratories, Inc., USA). Cell viability was calculated by the mean absorbance. Cell apoptosis was detected by Hoechst staining kit (Beyotime, China). After incubation for 24 h with Torin1 and lactate, NP cells were incubated in a 12-well cell culture plate with glass coverslips at 37°C, and the slides were stained by Hoechst 33258 according to instructions. The number of the apoptotic hyperchromatic nuclei and the total number of nuclei were counted in four microscopic areas. Cell counting was performed by ImageJ software (NIH, USA).

### Metabolomics analysis

Metabolic analysis was conducted in IL-1β- and Torin1- (100 nM) treated NP cells by GC-MS system (Agilent, USA). GC/TOFMS analysis was performed using an Agilent 7890 gas chromatograph system coupled with a Pegasus HT time-of-flight mass spectrometer (LECO Corp., USA). The system utilized a DB-5MS capillary column coated with 5% diphenyl cross-linked with 95% dimethylpolysiloxane. A 1-μL aliquot of the analyte was injected in splitless mode in standard producer. The mass spectrometry data were acquired in full-scan mode with the m/z range of 85-600 at a rate of 20 spectra per second after a solvent delay of 360 s. Chroma TOF4.3X software (LECO Corp.) and LECO-Fiehn Rtx5 database were used for raw peaks exacting, data baselines filtering and calibration of the baseline, peak alignment, deconvolution analysis, peak identification, and integration of the peak area. The RI (retention time index) method was used in the peak identification, and the RI tolerance was 5000.

### Western blots

NP cells were lysed on ice with ice-cold 2% SDS buffer. Equal amounts of protein were separated by SDS-PAGE and transferred onto polyvinylidene difluoride membranes (Millipore, USA). Non-fat 5% dried milk diluted in TBST for 1 h was used for blocking. The membranes were incubated overnight with primary antibodies and then visualized using HRP-conjugated secondary antibodies (Pierce, USA). Finally, proteins were detected by Super Signal^®^ enhanced chemiluminescence development (ECL, Pierce, USA) reagent and exposed by the Bio-Rad system.

### Real time-PCR assay

Total RNA of NP cells was extracted by Trizo (Invitrogen, USA) with a standard protocol and reversed transcribed by PrimeScriptTM RT reagent kit with gRNA Eraser (Takara, Japan). The results were analyzed by quantitative PCR (qPCR) using SYBR Premix Ex TaqTM II (Takara) and the Bio-Rad iQ5 system. A complete list of PCR primers is shown in Supplementary Table S1.

### Statistical analysis

Data are reported as means±SE. Statistical analysis was performed by two-tailed Student's *t*-test or one-way ANOVA tests for comparisons using GraphPad Prism 7.0 software (USA). A P value of <0.05 was considered statistically significant.

## Results

### Pro-inflammatory cytokine IL-1β increased mTORC1 activity in NP cells

**Figure 1:**
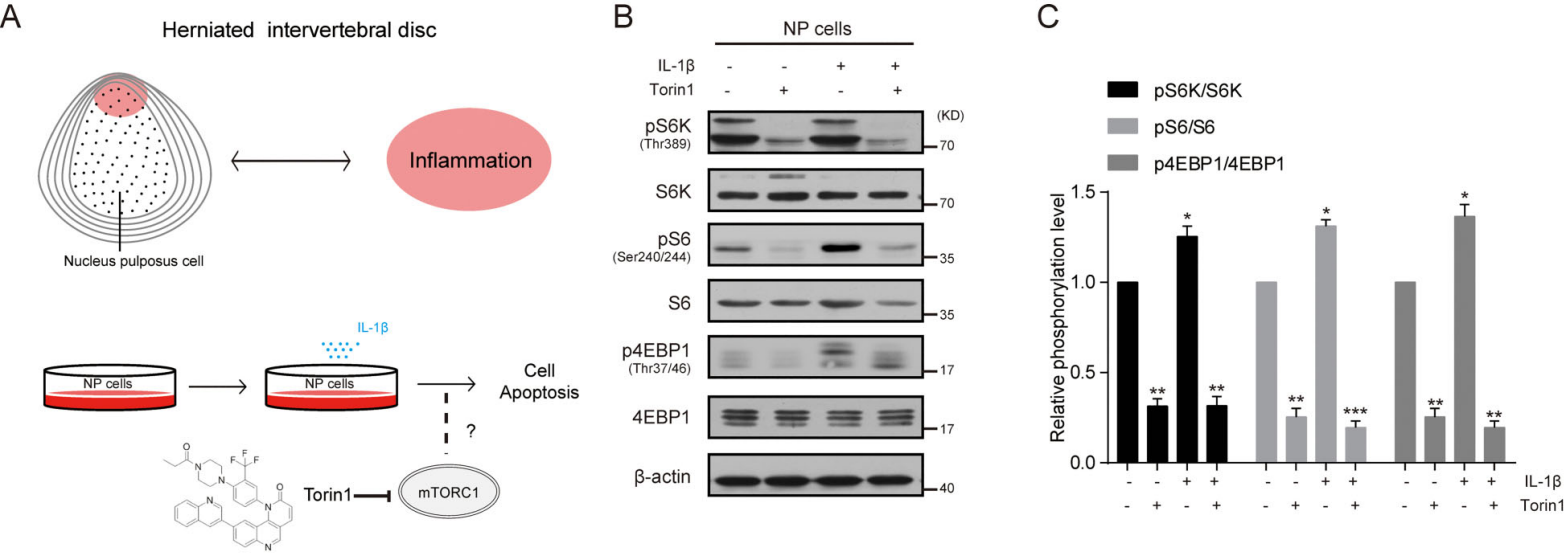
Torin1 treatment decreases interleukin (IL)-1β-induced mTORC1 activation in nucleus pulposus (NP) cells. **A**, A diagram showing the experimental design of this study to investigate a role of mTORC1 signaling in pro-inflammatory cytokine-induced NP cell apoptosis. **B** and **C**, Western blots and quantifications showing that IL-1β (10 ng/mL, 24 h) increased mTORC1 activity and Torin1 treatment (100 nM, 24 h) inhibited mTORC1 activity (indicated by pS6K, pS6, and p4EBP1 levels). Data are reported as means±SE for n=3. *P<0.05, **P<0.01, and ***P<0.001 compared to control (two-tailed one-way ANOVA with Dunnett *post hoc* test).

Pro-inflammatory cytokine IL-1β is closely linked to the pathogenesis of IVDs and increases with disease progression (12,22). mTORC1 signaling is a central regulator of cellular inflammation and metabolism, which is reported to be associated with LDH degeneration (17). Therefore, we first examined whether mTORC1 activity in human NP cells was affected by IL-1β (Figure 1A). Results of western blots showed that IL-1β increased mTORC1 activity and increased protein levels of pS6K, pS6, and p4EBP1. Moreover, we applied Torin1, a well-known mTORC1 inhibitor, to NP cells and found that Torin1 treatment significantly blocked mTORC1 activity induced by IL-1β. The total protein levels of S6K, S6, and 4EBP1 were not altered by either IL-1β or Torin1 treatment (Figure 1B and C). These results established the *in vitro* system to mimic IVD degeneration and suggested that Torin1 can block IL-1β-induced mTORC1 activity in NP cells.

### Torin1 inhibited mTORC1 activity and protected against the apoptosis of NP cells

**Figure 2:**
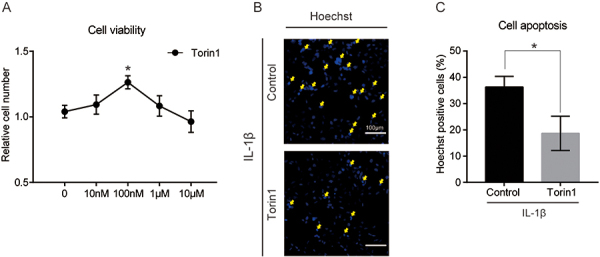
Torin1 treatment decreased interleukin (IL)-1β-induced apoptosis in nucleus pulposus (NP) cells. **A**, Quantification of relative cell numbers indicating cell viability by Torin1 treatment (24 h) in NP cells. **B** and **C**, Representative images and quantification of Hoechst staining indicating that Torin1 treatment (100 nM, 24 h) inhibited IL-1β-induced NP cell apoptosis. Yellow arrows indicate apoptotic cells. Scale bar, 100 μm. Data are reported as means±SE for n=3. *P<0.05 (two-tailed Student's *t*-test).

mTORC1 is a central regulator of cell growth, differentiation, and apoptosis. Results of CCK-8 assay showed that 100 nM Torin1 treatment slightly increased cell viability of IL-1β-treated cells, but decreased cell viability at higher concentrations (10 μM) (Figure 2A). Hoechst staining showed that the percentage of apoptotic-positive cells was significantly decreased by Torin1 treatment under IL-1β conditions (Figure 2B). Quantifications also confirmed the protective effect of Torin1 on NP cell survival (Figure 2C). These data support the fact that mTORC1 signaling was involved in inflammation-induced NP cell death.

### Inhibition of mTORC1 by Torin1 altered metabolic homeostasis of NP cells

**Figure 3:**
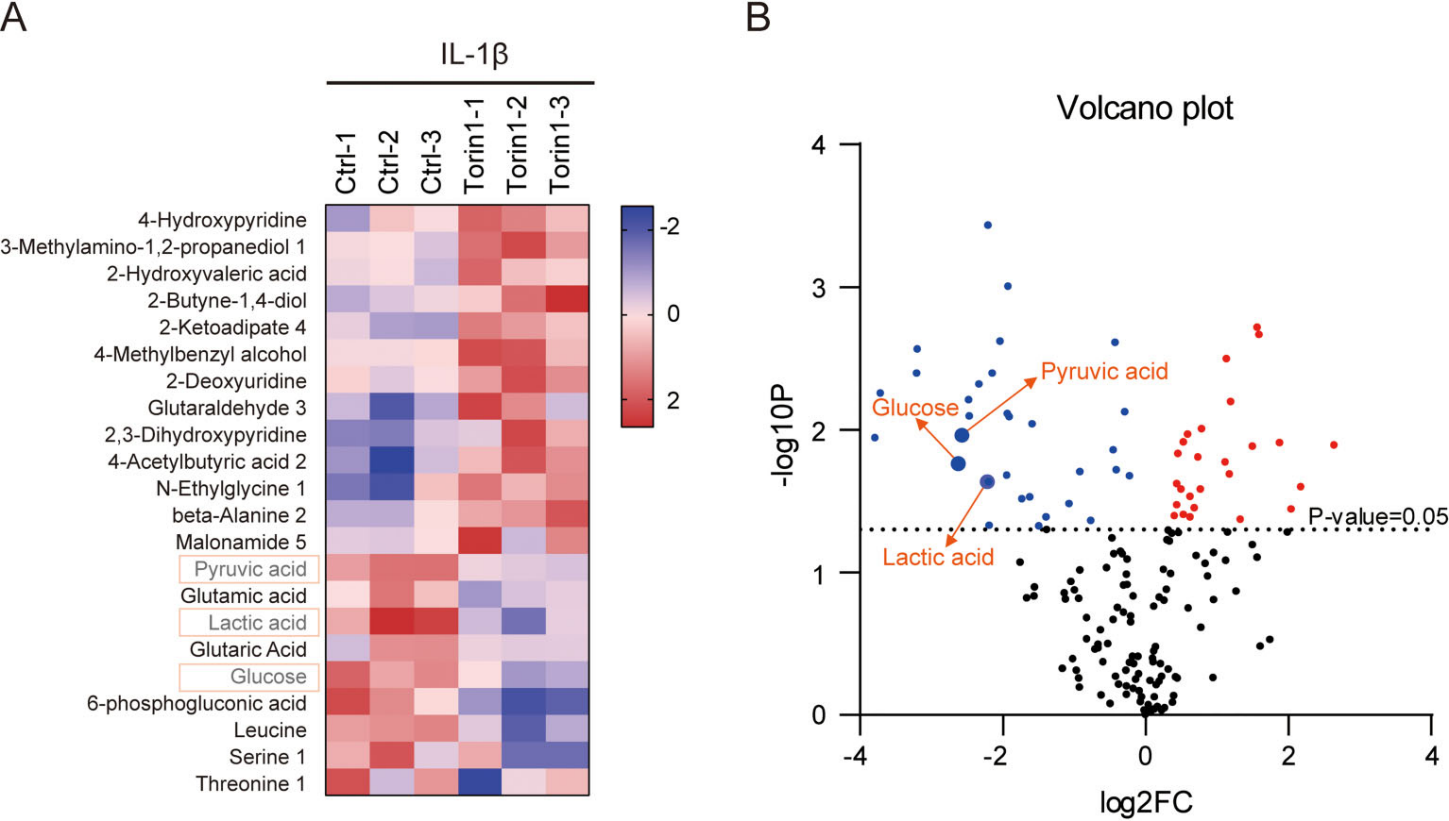
Inhibition of mTORC1 activity altered metabolic homeostasis in nucleus pulposus (NP) cells. **A**, Heatmap showing the selective altered metabolites in NP cells treated by Torin1 (100 nM, 24 h) under inflammation conditions. **B**, Volcano plots showing representative metabolites. Arrows indicate key metabolites in glucose metabolism, including glucose, pyruvic acid, and lactic acid.

Results of metabolomics showed that Torin1 can alter metabolite contents under IL-1β conditions in NP cells (Figure 3A). By Torin1 treatment, 12 metabolites were increased and 10 were decreased in IL-1β-treated NP cells. Metabolites of glucose metabolism, including glucose, pyruvic acid, and lactic acid, were significantly altered by Torin1 treatment by stringent selection (Figure 3B). Notably, we found that lactic acid level was significantly decreased by Torin1 treatment. Lactic acid is a product from aerobic glycolysis and participates in several metabolic processes in the body, such as inflammatory responses and cell survival (23). Hence, our data indicated that mTORC1 signaling may regulate NP cell survival/apoptosis by controlling lactic acid production.

### mTORC1 signaling regulated lactic acid levels and subsequent NP cell survival

**Figure 4:**
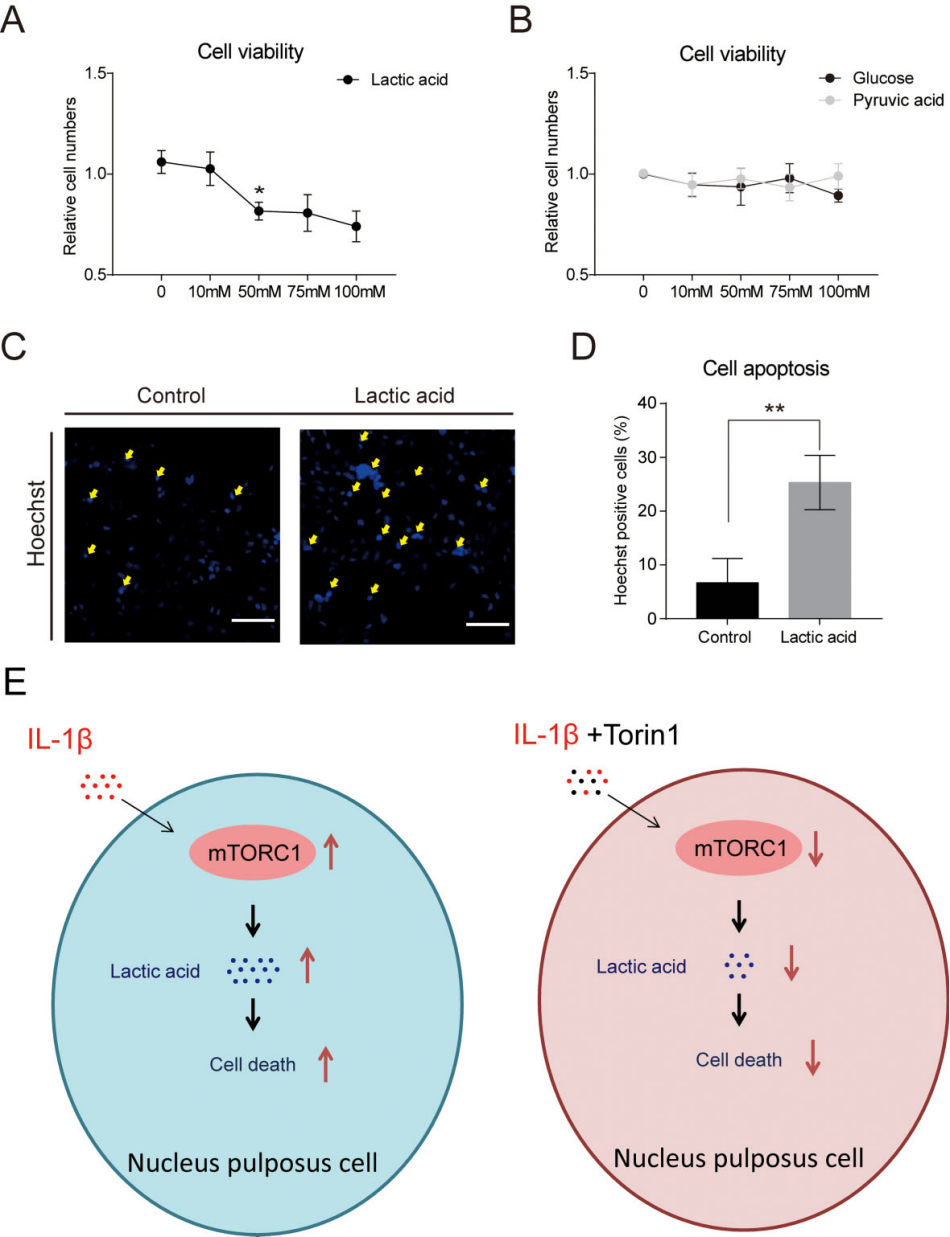
Torin1 inhibited nucleus pulposus (NP) cell apoptosis induced by lactic acid. **A**, Quantification of relative cell numbers indicating that lactic acid induced NP cell death (>50 mM, 24 h). **B**, Quantification of relative cell numbers indicating cell viability by glucose or pyruvic acid (24 h) treatment, respectively. **C** and **D**, Images (scale bar, 100 μm) and quantification of Hoechst staining showing lactic acid induced NP cell apoptosis. Data are reported as means±SE for n=3. *P<0.05 and **P<0.01, compared to control (two-tailed Student's *t*-test). **E**, Model illustrating that inhibition of mTORC1 activity protects against inflammation-induced apoptosis in NP cells.

To examine the role of mTORC1-controlled metabolites in NP cell survival, we conducted CCK-8 assay in NP cells stimulated by glucose/pyruvic acid/ lactic acid. Results showed that lactic acid decreased NP cell viability at a concentration of 50 mM, but glucose and pyruvic acid treatment did not alter NP cell viability (Figure 4A and B). Thus, we propose that lactic acid may affect NP cell survival under IL-1β conditions. Hoechst positive cells were significantly increased compared to controls, suggesting lactate treatment may negatively regulate NP cell survival (Figure 4C and D). Hexokinase is a rate-limiting enzyme of glycolysis (24), and lactate dehydrogenase is the enzyme converting pyruvic acid to lactic acid (25). We found that Torin1 treatment significantly decreased the gene expressions of hexokinase and lactate dehydrogenase (Supplementary Figure S1). Taken together, these results indicated that mTORC1 signaling regulated lactic acid production and thus controlled NP cell survival.

## Discussion

Degeneration of IVDs is significantly associated with tissue inflammation and metabolic imbalance (12), but the molecular mechanism of IVD degeneration is not clear. Our study demonstrated that mTORC1 signaling regulated inflammation-induced NP cell apoptosis in a metabolic manner. Inhibition of mTORC1 activity protected against NP cell apoptosis (Figure 4E).

Currently, it is known that increased NP cell death is a fundamental mechanism of IVD degeneration and LDH progression, with inflammatory cytokines release (26). A previous study reports that mTORC1-hyperactivation under stress conditions can induce cell death (27). In the present study, we revealed that released inflammatory cytokine enhanced mTORC1 activity in NP cells, leading to cell apoptosis. We showed that mTORC1 activation impaired the metabolic homeostasis and contributed to the inflammation-induced cell death of NP cells. mTORC1 activity is significantly associated with cell metabolism. Studies report that glutamine degradation by glutaminolysis increases mTORC1 pathway and thus inhibits autophagy and induces apoptosis in cancer cells (28). Our study provided additional evidence that mTORC1 is also involved in glucose/lactate metabolism and participated in metabolite-controlled cell death. Moreover, we conducted pharmacological inhibition of mTORC1 activity to rescue inflammation-induced NP cell death. This work suggested a new clue for clinical interventions of LDH.

LDH is developed with anatomic, morphologic, biochemical, and metabolic changes. mTORC1 signaling can integrate extracellular stimulation and intracellular responses to modulate cell proliferation and apoptosis (18). Inhibition of mTORC1 activity impairs cellular energy metabolism by reducing glucose utilization through glycolysis. Here, we showed that Torin1 treatment decreased glucose, pyruvic acid, and lactic acid levels, indicating that part of carbohydrate metabolism was disrupted. Interestingly, the final product of aerobic lactate glycolysis is significantly associated with inflammation responses (23). Our work established the metabolic axis of inflammation/mTORC1/lactate in the precise regulation of NP cell survival/death, which supplements current understandings on NP cell biology. Future work should be directed at inhibition of mTORC1 activity *in vivo* by genetic or pharmacological methods to test whether mTORC1 inhibition may be beneficial to ameliorate inflammation-induced NP cell death and IVD degeneration.

In conclusion, this study revealed a novel mechanism by which mTORC1 signaling controls NP cell survival through regulating lactic acid production. Our findings identified mTORC1 in NP cells as a novel target for IVD degeneration, providing potential strategies for LDH intervention.

## Supplementary Material

Click here to view [pdf].
